# rTMS Induced Tinnitus Relief Is Related to an Increase in Auditory Cortical Alpha Activity

**DOI:** 10.1371/journal.pone.0055557

**Published:** 2013-02-04

**Authors:** Nadia Müller, Isabel Lorenz, Berthold Langguth, Nathan Weisz

**Affiliations:** 1 Università degli Studi di Trento, Center for Mind/Brain Sciences, Mattarello, Italy; 2 University of Konstanz, Department of Psychology, Konstanz, Germany; 3 University Hospital Regensburg, Department of Psychiatry and Psychotherapy, Regensburg, Germany; 4 Zukunftskolleg, University of Konstanz, Konstanz, Germany; Université catholique de Louvain, Belgium

## Abstract

Chronic tinnitus, the continuous perception of a phantom sound, is a highly prevalent audiological symptom. A promising approach for the treatment of tinnitus is repetitive transcranial magnetic stimulation (rTMS) as this directly affects tinnitus-related brain activity. Several studies indeed show tinnitus relief after rTMS, however effects are moderate and vary strongly across patients. This may be due to a lack of knowledge regarding how rTMS affects oscillatory activity in tinnitus sufferers and which modulations are associated with tinnitus relief. In the present study we examined the effects of five different stimulation protocols (including sham) by measuring tinnitus loudness and tinnitus-related brain activity with Magnetoencephalography before and after rTMS. Changes in oscillatory activity were analysed for the stimulated auditory cortex as well as for the entire brain regarding certain frequency bands of interest (delta, theta, alpha, gamma). In line with the literature the effects of rTMS on tinnitus loudness varied strongly across patients. This variability was also reflected in the rTMS effects on oscillatory activity. Importantly, strong reductions in tinnitus loudness were associated with increases in alpha power in the stimulated auditory cortex, while an unspecific decrease in gamma and alpha power, particularly in left frontal regions, was linked to an increase in tinnitus loudness. The identification of alpha power increase as main correlate for tinnitus reduction sheds further light on the pathophysiology of tinnitus. This will hopefully stimulate the development of more effective therapy approaches.

## Introduction

Tinnitus is defined as the subjective perception of a sound in the absence of any physical sound source. If persisting longer than a certain amount of time, conventionally between six and twelve months, it is usually regarded as ‘chronic’, reflecting clinical experience that the phantom sound will persist. Chronic tinnitus is a common phenomenon with a prevalence of 5–15% of the population in western societies [1], [2]. In 1–3% of the population, tinnitus is associated with severe distress including psychiatric problems (e.g., depression), sleep disturbances, concentration problems or work impairment [1]. To date, there is no effective treatment that reliably eliminates tinnitus [1], partly because the processes that generate and maintain tinnitus and its associated problems are not completely understood. A broad consensus, however, is that tinnitus is generated in central brain structures rather than in the peripheral auditory system. Evidence comes from clinical studies showing that the tinnitus percept persists even after transection of the auditory nerve fibres [3], [4].

In most cases, tinnitus is associated with a damage of hair cells in the inner ear [5], [6], resulting in pathological neuronal activity in central structures [7]–[10]. Various neurophysiological processes at different levels of the auditory system that are elicited by hearing loss have been suggested as being involved in the generation of tinnitus [9]. Hearing loss, for instance, results in a loss of inhibition and a reorganisation of the tonotopic map [11], [1]. Studies in animals and humans demonstrate that the tinnitus sensation is associated with hyperactivity in subcortical and cortical auditory brain structures. This hyperactivity is reflected in an enhanced spontaneous firing rate [12]–[14], elevated bursting activity [13], [15] and increases in neural synchrony that have been shown to correspond closely to hearing loss [16]. Roberts et al. (2010) postulate that, among these processes, the increase in neural synchrony seems to be most relevant for the actual generation of tinnitus as it has the potential to impact postsynaptic targets and recruit cortical and downstream neurons into a tinnitus percept. The role of altered synchrony in tinnitus is strongly supported by studies that report changes in oscillatory brain activity associated with tinnitus [7], [17]–[19] on a subcortical and cortical level. On a subcortical level, for instance, abnormal low-frequency activity in the thalamus can lead to disturbances in the thalamo–cortico–thalamic network (thalamocortical dysrhythmia, TCD) and thereby influence perception [20]. On the cortical level it has been shown that oscillatory activity in the so-called alpha band (8–12 Hz), which has been related to inhibitory processes [21], is reduced in the auditory cortex of tinnitus patients [10]. Power increases were found for low frequencies in the delta [10] to theta range [20], [22], [23], [24] and in gamma power compared to normal hearing controls [7], [18], [25].

A promising treatment approach for chronic tinnitus is transcranial magnetic stimulation (TMS) [26], [27] as this affects brain activity directly, thereby holding the potential to influence abnormal ongoing brain activity related to tinnitus. Particularly in its repetitive form (rTMS; [28], [29]), it has been shown (mostly in the motor system) that stimulation-induced changes of excitability and plasticity outlast the period of stimulation. A growing number of studies indeed point to tinnitus relief after a series of ten rTMS sessions (for an overview see [30]), with effects lasting for up to four years [31]. However, the effects show great interindividual variability [27], [32] and only moderate effect sizes [33]. Only few studies have investigated the effects of rTMS on auditory cortical activity in tinnitus sufferers and which aspects of these modulations are relevant for tinnitus relief. In a previous study, we were able to demonstrate that various forms of single session rTMS (particularly cTBS, iTBS and 1 Hz rTMS) could reduce the auditory Steady State Response (aSSR), which is in turn correlated with subjectively perceived tinnitus loudness [34]. In the context of the same study we also collected resting MEG activity. To date, no published reports have investigated the impact of rTMS on spontaneous oscillatory brain activity in tinnitus patients - a potentially fundamental element in the generation of tinnitus [9], [10]. In order to better understand the pathophysiology of tinnitus and also to systematically advance tinnitus therapies, it is essential to know if and how oscillatory brain activity in tinnitus patients is modulated by rTMS and what changes in oscillatory activity are crucial for tinnitus suppression. This view is also strongly supported by Fuggetta and colleagues who emphasized that we will gain further insight into therapy approaches and the pathophysiology of a variety of neurological disorders by investigating rTMS effects on TCD-like EEG patterns [23]. With the current data we are able to show - on the level of group as well as single subject statistics - that tinnitus relief after rTMS is associated with an increase in alpha activity in the auditory cortex, thus supporting the relevance of alpha activity for tinnitus [10].

## Methods

### 1. Participants

Ten patients with chronic tinnitus participated in the current study (7 males, 3 females). The mean age of the participants was 49.8 years (range: 21–70). Patients were recruited via advertisements in the local newspaper and flyers posted at the University of Konstanz. Tinnitus severity was assessed with Hallam’s [35] Tinnitus Questionnaire (*Tinnitus-Fragebogen*; [36]), revealing a mean score of 29.9 (range: 8–59). Half of the patients reported unilateral tinnitus (4 with left-sided tinnitus, 1 with right-sided tinnitus), while the other half indicated having perceived the tinnitus bilaterally. We only included patients with a maximum tinnitus duration of four years, as the impact of rTMS on chronic tinnitus declines with longer tinnitus duration [26]. All patients were informed about the content of the study as well as the potential risk factors and underwent a thorough anamnesis concerning potential contraindications for TMS (previous personal or family history of epileptic seizures, cardiac pacemaker, pregnancy, neurodegenerative diseases, brain injuries). Furthermore, patients with psychiatric or neurological disorders according to the M.I.N.I. (Mini International Neuropsychiatric Interview, German Version 5.0.0) and with anticonvulsant or tranquilizer medication were excluded from the study. The Ethics Committee of the University of Konstanz approved the experimental procedure and the participants gave their written informed consent prior to taking part in the study.

### 2. Experimental Design

**Figure 1:**
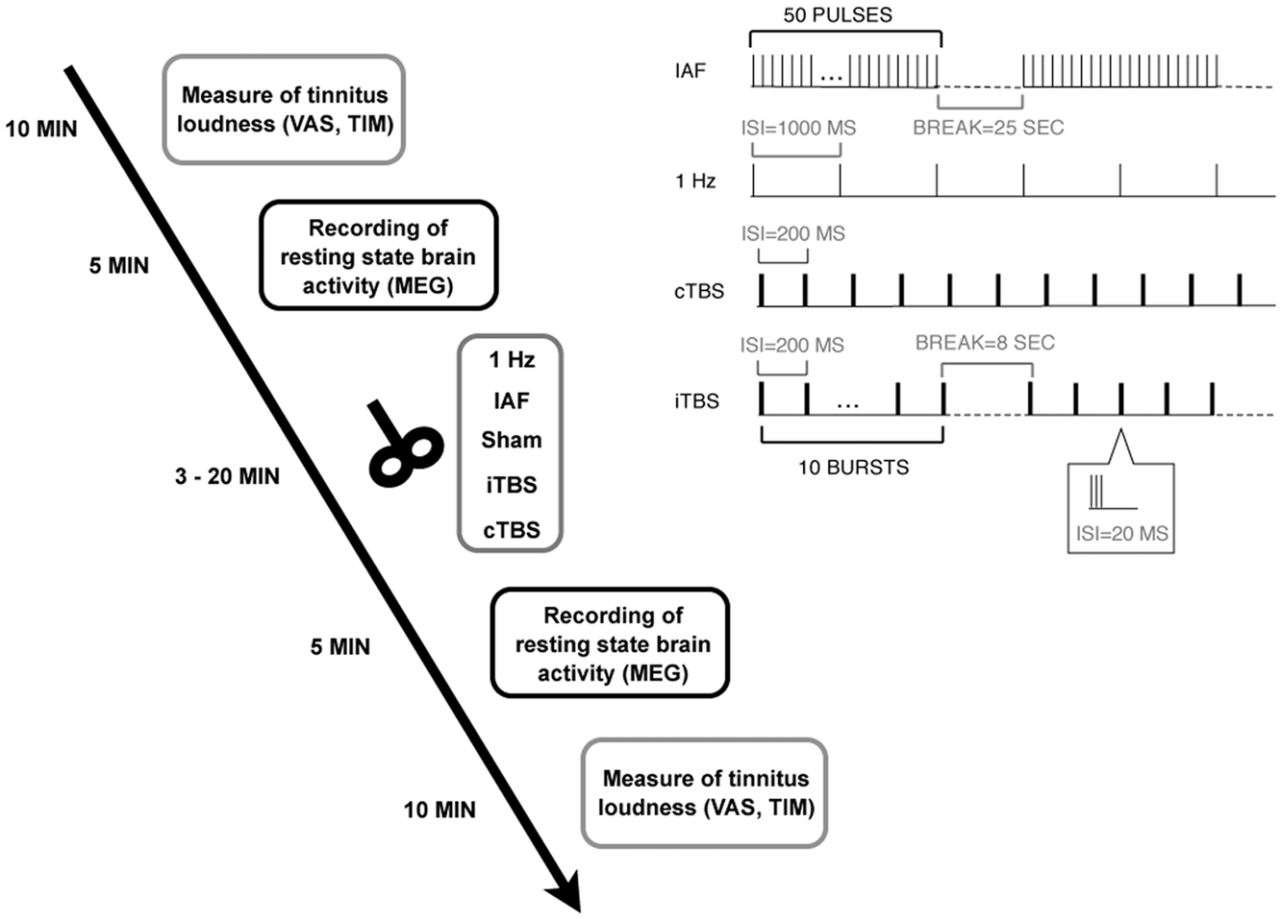
Experimental design. Patients underwent five sessions with five different rTMS protocols (including Sham). In each session, tinnitus loudness and oscillatory brain activity were measured before and after rTMS. The right upper panel illustrates the different stimulation protocols.

Patients underwent five sessions of rTMS, including the measurement of tinnitus loudness and brain activity with MEG before and after rTMS, resulting in a dataset of 100 MEG and 100 tinnitus loudness measurements (10×5×2). In the five sessions, five different rTMS protocols were applied, with a minimum interval of one week between sessions and using a randomized, single-blind, placebo-controlled design. For an overview of the study design, see Figure 1.

### 3. Measurement of Tinnitus Loudness

Before the first and after the second MEG recording, patients were asked to match the loudness of their tinnitus to a reference tone of 1 kHz (tinnitus intensity matching; TIM). This procedure considers the absolute hearing threshold of the 1-kHz tone so that the matched tinnitus intensity is expressed in ‘sensation level’. Additional to this psychoacoustic assessment, the patients estimated their perceived tinnitus loudness on a visual analogue scale (VAS) ranging from 0 (‘not loud at all’) to 10 (‘extremely loud’).

### 4. Data Acquisition with MEG

The MEG recordings were carried out using a 148-channel whole-head magnetometer system (MAGNESTM 2500 WH, 4D Neuroimaging, San Diego, USA) installed in a magnetically shielded chamber (Vakuumschmelze Hanau). Prior to the recording, individual head shapes were collected using a digitiser. Participants lay in a supine position and were asked to keep their eyes open and to focus on a fixed point on the ceiling during the recording. The recording time was five minutes. MEG signals were recorded with a sampling rate of 2034.51 Hz and a hard-wired high-pass filter of 0.1 Hz. MEG measurements were conducted before and after TMS. The time interval between the end of the TMS session and the start of the second MEG recording did not exceed five minutes.

### 5. Brain Stimulation with TMS

TMS stimulation (biphasic magnetic pulses) was administered with a figure-of-eight coil (coil winding diameter 2×75 mm; Magnetic Coil Transducer C-B60, Medtronic) connected to a MagPro X 100 TMS stimulator (Medtronic A/S, Skovlunde, Denmark).

Five different stimulation protocols were applied in randomized order over five sessions separated by at least one week: 1-Hz rTMS (1 train with 1000 pulses, frequency 1 Hz), individual alpha frequency rTMS (IAF; 20 trains with 50 pulses and 25 seconds inter-train interval, peak frequency ranging between 8 and 12 Hz), continuous theta burst stimulation (cTBS; 200 bursts at a frequency of 5 Hz with bursts consisting of 3 pulses at 50 Hz), intermittent theta burst stimulation (iTBS; 10 trains of 10 bursts at a frequency of 5 Hz with bursts consisting of 3 pulses at 50 Hz and an 8 seconds inter-train interval), and a placebo sham stimulation (45° coil angulation, applying the IAF protocol). Individual alpha frequency was defined as the peak of the power spectrum (between 8 and 12 Hz) at temporal sensors in the first MEG recording. For an illustration of the different protocols see right upper panel of Figure 1. The patients were blind to the TMS condition. The intensity of the stimulation was adjusted according to the resting motor threshold (RMT) –a common procedure in TMS studies [37]. RMT was measured by delivering single pulses at the optimal site over the motor cortex and defined as the lowest stimulation intensity required for producing visible hand muscle contractions in at least five out of ten trials as it has been done in previous studies [37]. For 1-Hz rTMS, IAF, and sham stimulation, intensity was set to 110% of the RMT and for iTBS and cTBS to 80% of the RMT. Thus, the intensities we applied were slightly higher than those used by Huang et al. [87] for motor cortex stimulation. To prevent hearing damage caused by the loud clicking sound of the TMS device, patients were required to use earplugs. Patients were seated in a comfortable chair while the TMS coil was fixated with a mechanical arm. The handle of the coil always pointed upwards. In case of right-ear or bilateral tinnitus, the coil was placed over left Heschl’s gyrus by moving 2.5 cm upwards from T3 on the line between T3 and Cz and then 1.5 cm perpendicularly in a posterior direction, analogously over right Heschl’s Gyrus in case of left-ear tinnitus. This procedure has been proven to reliably position the TMS coil over the auditory cortex [38].

### 6. Data Analysis

#### 6.1. Preprocessing

We analysed the data sets using Matlab (The MathWorks, Natick, MA, Version 7.5.0 R 2007b) and the Fieldtrip toolbox [39]. We separately extracted two-second epochs from the continuously recorded MEG signal for the measurements, resulting in 150 trials for the pre (baseline) and post-TMS condition, respectively. We then performed artefact rejection in two steps. First, we visually inspected trials for eye movements, muscle artefacts or channel jumps and rejected the affected trials. Furthermore, we eliminated dead and very noisy channels. Two out of 100 data sets (one from the cTBS and one from the IAF protocol) had to be excluded because of very poor data quality. In a second step, the data sets were processed using an Independent Component Analysis (ICA; http://sccn.ucsd.edu/eeglab/) to correct for heartbeat-related artefacts. We entered 80 randomly sampled trials into the ICA in order to get independent components with a distinct time course and spatial topography. We identified those components (two in the majority of cases) that captured cardiac activity through visual inspection. Afterwards, the respective weights of the ICA were applied to the whole data set, artefact components were removed and the original data were reconstructed without the impact of the artefact. To ensure similar signal-to-noise-ratio for direct comparisons between the placebo (sham) and active TMS conditions, the trial number was adjusted to the minimum remaining trial number for the two time points (pre and post) and the compared conditions (sham and the respective active TMS protocols). To keep trial numbers in a comparable range, maximum trial number was set to 90.

#### 6.2. Spectral power analyses derived from auditory cortex

As patients had to leave the MEG between pre and post-TMS sessions, all analyses were performed at source level in order to obtain robust effects, in contrast to a potential analysis at sensor level, which would have been more susceptible to altered head positions in the sensor helmet (from pre to post as well as over different days).

For each patient, we created a head model fitted to the head shape of the first MEG measurement using a multisphere approach [40]. This yielded a grid covering the entire brain with a resolution of 1 cm and assured that the same grid would be used in a single participant across all sessions. The leadfield for each grid point, however, was separately calculated for each session to account for potentially altered positioning of the sources with respect to the sensors.

Data were then analysed for the region of interest, defined as the auditory cortex (Brodman Area 41 & 42; Talairach atlas) ipsilateral to the TMS stimulation side. We also investigated oscillatory brain activity contralateral to the stimulation side. This analysis did not reveal any consistent effects; we thus do not describe them in further detail. In order to estimate power spectra for the region of interest, we employed a multitaper spectral estimation method [41] to the ICA-corrected raw data and kept the complex Fourier coefficients. We used a different smoothing for low (2–12 Hz) and high frequencies (30–90 Hz) so that the data were multiplied with a set of orthogonal Slepian tapers, yielding a frequency smoothing of +/−1 Hz for low and +/−5 Hz for high frequencies. We then constructed spatial filters (with fixed orientation) using the lcmv-algorithm (lcmv beamformer; [42]) for each grid point within the region of interest. This was again accomplished for low and high frequencies respectively by filtering the non-ICA corrected data in the corresponding frequency bands (2–12 Hz, 30–90 Hz). Afterwards, we projected the complex values into source space by multiplying them with the accordant spatial filters and by calculating the complex modulus of the values. We thereby obtained absolute power values for each voxel within the region of interest. By averaging the values in the region of interest we obtained one single value for each frequency. This procedure was repeated for each patient, for both time points (pre and post) and for the five different conditions (4 active TMS protocols & sham). Finally, spectral source estimates were normalized for each patient and condition by calculating a (post-pre)/pre ratio, reflecting the modulation of oscillatory power from pre to post TMS intervention. It should be noted that we focused on frequencies of interest that were derived from previous studies on altered auditory oscillatory power in tinnitus patients: delta (1–3 Hz; [10]), theta (4–6 Hz; [20], [22]; alpha (8–12 Hz; [10]), gamma ([7], [18], [25]) subdivided in low gamma (30–70 Hz) and high gamma (70–90 Hz). In the next step, these values were statistically tested.

#### 6.3. Statistical analyses of the pre-post modulations

Statistical analyses were performed using R version 2.11.1 for Mac OS X (www.r.project.org). As the complex study design entailed a small sample size and we identified various ‘outliers’ that, after precise investigation, we did not wish to treat as real outliers, we used a bootstrap approach for the statistical analysis. The ‘outliers’ or extreme values were not due to poor data quality, but rather reflect strong interindividual differences in the different stimulation protocols with somehow systematic patterns (only for specific TMS protocols and in patients with very short tinnitus duration; see Figure S1 in the supplemental material for comparison). Therefore, we decided not to exclude these cases and instead use robust statistics. We always compared the pre-post ratios of the active TMS conditions against sham using a bootstrap approach (‘boot’ package included in R). Thus, the sham values were subtracted from the activation values for each patient. After that we created 1000 new samples from the original sample (by drawing with replacement). For each of these new samples (having the same size as the original sample) the median was calculated. We thereby obtained a distribution of 1000 bootstrapped medians. The median was used in order to not overemphasize extreme values. We subsequently extracted the 95% confidence intervals (CI) for the median for each stimulation form respectively. If the confidence interval did not include 0, the effect could be considered as significant (power modulation significantly different in the active TMS vs sham TMS condition).

We performed this procedure for both, the modulation of tinnitus loudness and the modulation of auditory oscillatory activity in the frequency bands of interest.

#### 6.4. Signatures of auditory brain activity reducing tinnitus loudness

Apart from analyses that focused on consistent modulations of tinnitus loudness and oscillatory activity after the different TMS protocols, we wanted to identify the signatures of oscillatory brain activity that are decisive for a strong reduction or an enhancement of tinnitus loudness. Hence, we defined the most effective stimulation protocol (among active TMS conditions) in *reducing* tinnitus loudness according to the TIM scores for each patient and analysed the according modulations in oscillatory activity. We repeated this procedure for the stimulation protocols that *increased* tinnitus loudness. For the selection of the according TMS protocol we used the TIM scores as they clearly separated the different protocols, whereas the VAS scores were more ambiguous (i.e., different protocols lead to identical modulations of tinnitus loudness). Note that we obtained similar results when excluding the ambiguous cases in the VAS assignment compared to the TIM assignment.

We conducted a further bootstrap statistic (the same method as described above) for the five frequency bands of interest (delta, theta, alpha, low gamma, high gamma) and for both increasing and decreasing tinnitus loudness protocols. We thereby defined the signatures of neuronal activity resulting in tinnitus loudness decreases/increases within the same participants.

#### 6.5. Auditory alpha power modulation for the individual patients

The group results showed a high inter-individual variance for all investigated stimulation protocols, but also hinted at the potential of rTMS for treating tinnitus when it is applied in an individually optimized way (i.e. selecting protocol that increases auditory alpha power, see results section; see also Discussion indicating that this issue needs more definite confirmation). In order to address the clinically highly relevant question of the neuronal changes on an individual level we decided to add a single subject analysis. This was done to elucidate the pattern of how auditory alpha activity is modulated in the individual patients by the different stimulation protocols, in order to obtain information how the high variability within the protocols is made up. We therefore repeated the same analysis as described in the section on ‘*Spectral power analyses derived from auditory cortex’,* focussing on alpha power (as this was the most illuminating frequency band based on the group level results; see results section) however this time projecting the complex fourier spectra of the *single trials* onto our sources of interest. We then performed a single patient statistic by comparing the pre-post ratios of the single trials for each patient and TMS condition separately. Therefore, 5000 bootstrap replicates of the median were generated. We subsequently extracted the upper and lower quantiles corresponding to a probability of 5% and obtained the confidence intervals (CI) for each patient and stimulation form, respectively. This bootstrap procedure was in line with the one described above. Note that we here did not control for multiple comparisons so that the results should be interpreted on an explorative level only.

In a second step, we defined the most effective stimulation protocol in *reducing* tinnitus loudness according to the TIM (as already described for the group level in the section on ‘*Signatures of auditory brain activity reducing tinnitus loudness*’) for each patient separately and performed a single patient statistic quantifying the auditory alpha power modulation for the selected stimulation protocols. We further related the extent of the auditory alpha power modulation to the extent of the tinnitus loudness reduction (ranked from 1 to 10 with 1 reflecting the strongest loudness reduction). This procedure was repeated for the stimulation protocols that increased tinnitus loudness.

#### 6.6. Signatures of whole brain activity reducing tinnitus loudness

Although it was not the focus of the present study, we examined the signatures of oscillatory brain activity in non-auditory regions that are decisive for a strong decrease or increase in tinnitus loudness. We thus performed a whole brain analysis for the stimulation protocol (individually selected) that most effectively reduced or enhanced tinnitus loudness according to the TIM and analysed power modulations from pre to post-TMS in the frequency bands of interest (delta, theta, alpha, low gamma, high gamma). For this purpose, we performed Dynamic Imaging of Coherent Sources (DICS), introduced by Gross and colleagues [43]. This beamformer technique optimally estimates the power for a certain location while suppressing activity at all other locations. The headmodel and leadfield were taken from the prior ROI analysis. For each grid point, we constructed a spatial filter from the cross-spectral density matrix of the MEG signal (not ICA-cleaned) at the frequency of interest (delta, theta, alpha, low and high gamma) and the respective leadfield. Thereafter we applied the spatial filters to the Fourier-transformed ICA-cleaned data (multitaper analysis) for the frequency of interest and divided the values by an estimate of the spatially inhomogenous noise (obtained for each gridpoint on the basis of the smallest value of the covariance matrix) in order to normalise this across participants. Afterwards we interpolated the resulting activation volumes to the individual MRI of the patients and normalised them to a template MNI brain provided by the SPM2 toolbox (http://www.fil.ion.ucl.ac.uk/spm/software/spm2). For statistical analysis, we calculated (post-pre)/pre ratios for each voxel of the source solutions respectively and tested these relative values against zero by applying a voxel-wise t-statistic. To correct for multiple comparisons, we defined a minimum cluster size (minimum number of neighbouring voxels above a given threshold that are required for a significant cluster) with AlphaSim provided by the Afni Package (http://afni.nimh.nih.gov/afni/doc/manual/AlphaSim.pdf). We thereby preserved the main non-auditory regions (>770 voxels) that were modulated by an effective tinnitus loudness-reducing or enhancing TMS stimulation in the frequency bands of interest.

## Results

### 1. Individual Tolerance of the TMS Stimulation

None of the patients showed serious side effects of rTMS apart from transient mild to moderate discomfort due to muscle contractions, involuntary movements of the jaw and cutaneous sensations during TMS stimulation. One patient experienced a mild headache after stimulation, which disappeared without medication after several hours. Another patient reported periods of complete absence of the tinnitus lasting for several minutes after 1 Hz rTMS. Three patients reported a worsening of their tinnitus after IAF stimulation lasting for several hours up to a few days.

### 2. Tinnitus Loudness Modulations for the Different Stimulation Protocols Compared to Sham

**Figure 2:**
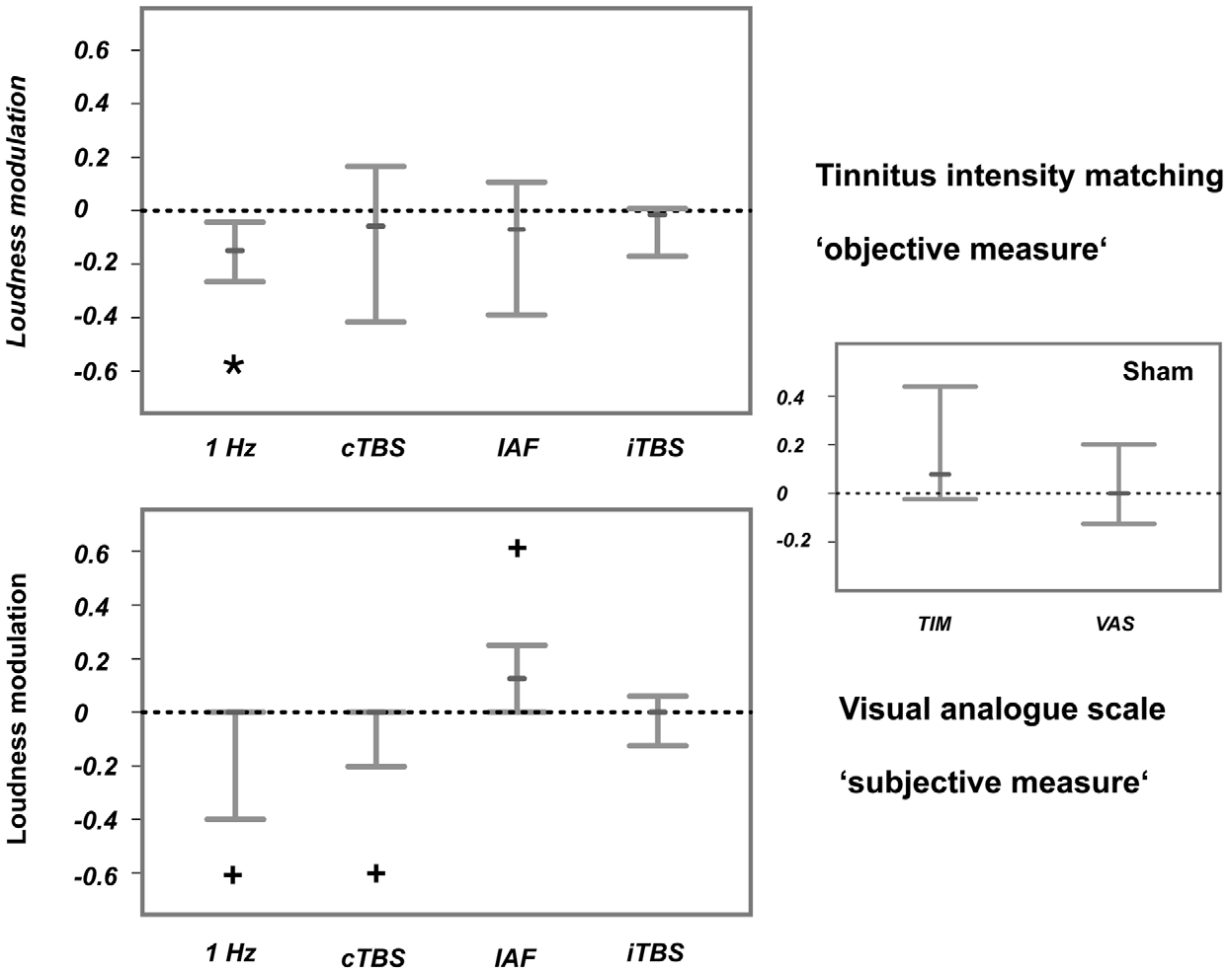
Consistent changes in tinnitus loudness after the four active TMS protocols compared to sham. The upper panel displays tinnitus loudness modulations that were measured with a matched-intensity approach (TIM), while the lower panel illustrates tinnitus loudness modulations that were quantified with a visual analogue scale (VAS). Sham effects are visualised in the right panel. Shown are the 95% confidence intervals. The small bars display the median. The asterisk indicates significant modulations, while the cross points to marginally significant modulations. According to the TIM, tinnitus loudness was reduced after 1-Hz rTMS. A trend pointing to a tinnitus reduction was revealed after 1-Hz rTMS and cTBS, while tinnitus loudness was marginally enhanced after IAF rTMS.

Matched tinnitus loudness (TIM) was significantly reduced for 1-Hz rTMS (median: −.15, 95% CI: −.04 to −.27) and not modulated for the other stimulation protocols (Figure 2; upper panel). The reduction of subjective tinnitus loudness (VAS) was marginally significant for the 1-Hz (95% CI: −0.4 to 0) and cTBS protocols (95% CI: −.25 to 0), whereas it turned out to be marginally enhanced for the IAF stimulation (95% CI: 0 to.26). ITBS did not consistently change the tinnitus loudness (see Figure 2 (lower panel) for comparison). TIM (median:.08) and VAS (median:.06) values were not significantly modulated by sham stimulation.

### 3. Modulations of Auditory Oscillatory Brain Activity for the Different Stimulation Protocols Compared to Sham

**Figure 3:**
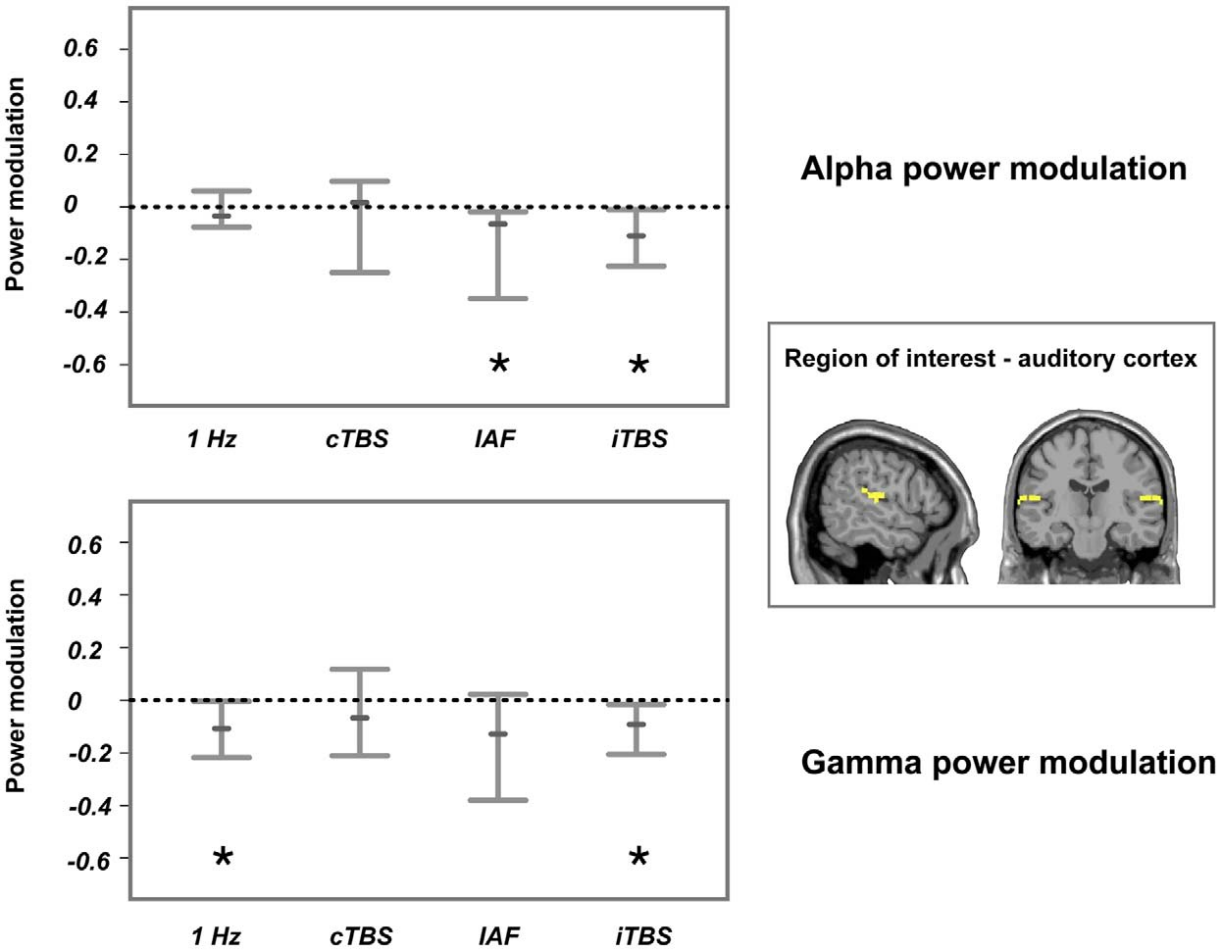
Consistent changes in oscillatory activity after the four active TMS protocols compared to sham. The upper panel displays alpha power modulations, while the lower panel illustrates modulations of high gamma power at the stimulated auditory cortex. The stimulated region and region of interest are displayed on the right side. Shown are the 95% confidence intervals. The small bars display the median while the asterisks indicate that the modulations were significant. Alpha power was significantly reduced after IAF rTMS and iTBS, while gamma power was significantly decreased by 1-Hz rTMS and iTBS (uncorrected).

Auditory oscillatory activity was not consistently modulated for the delta (1–3 Hz), theta (4–6 Hz) and low gamma (30–70 Hz) frequency bands (data shown in Figure S2 in the supplemental material)–that is, the confidence interval of all bootstrap statistics crossed the zero line. In contrast to this, power modulations in the alpha band were significantly reduced for the IAF stimulation (median: −.07, 95% CI: −.54 to −.02) and iTBS (median: −.10, 95% CI: −.20 to −.004), while no consistent differences were apparent in the cTBS and 1-Hz protocols (see Figure 3 upper panel). Furthermore, oscillatory power in the high gamma band (70–90 Hz) was significantly reduced for 1-Hz rTMS (median: −.11, 95% CI: −.24 to −.004) and iTBS (median: −.10, 95% CI: −.21 to −.02), while no consistent differences were apparent in cTBS and IAF stimulation (Figure 3, lower panel). Note, that we did not control for multiple comparisons, so that the results should be interpreted at an explorative level only.

Power modulations after sham stimulation were not significant (median of alpha power: .08; median of high gamma power.06).

### 4. Signatures in Auditory Oscillatory Power that Result in Strong Modulations of Tinnitus Loudness

**Figure 4:**
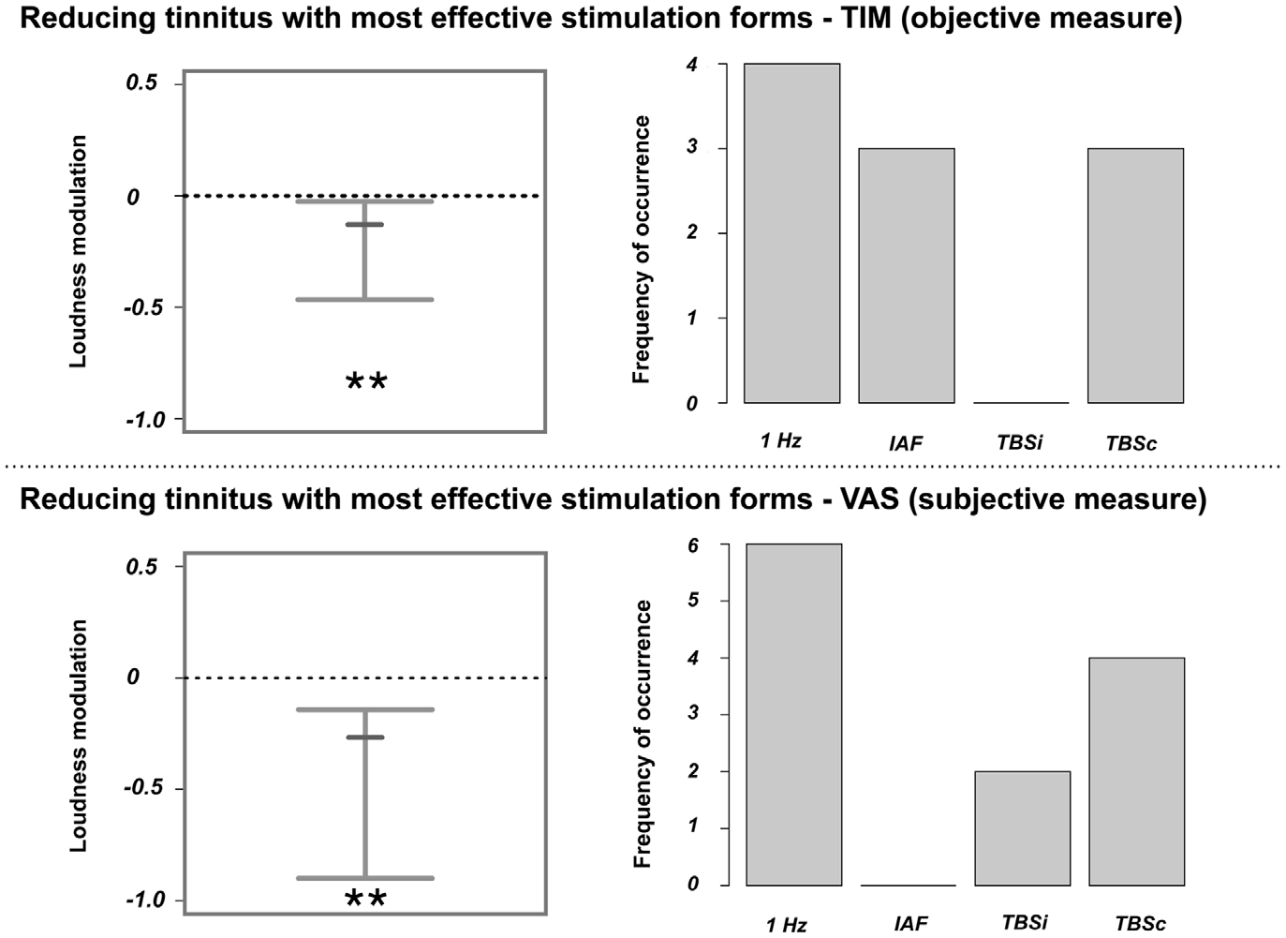
Changes in tinnitus loudness after application of the individually most effective stimulation protocol. The upper panel displays tinnitus loudness modulations that were measured with the tinnitus intensity matching procedure (TIM), while the lower panel illustrates tinnitus loudness modulations that were quantified with a visual analogue scale (VAS). The 99% confidence intervals are shown on the left side. The small bars display the median. The asterisks indicate significant modulations. As expected tinnitus loudness was significantly reduced after application of the individually selected protocol that was best in reducing tinnitus. The distribution of these protocols is displayed on the right side. Note that, as the ambiguous cases (when selecting the most effective protocol with VAS) were included for this illustration, the summed frequency of occurrence can be higher than the total number of patients.

Selecting the stimulation protocol (only active TMS protocols were considered, not sham) that was best in reducing tinnitus loudness for each patient individually resulted in a strong tinnitus reduction from pre to post rTMS for both the subjective (VAS; median: −.27, 99% CI: −.9 to −.14, only unambiguous cases included) and objective (TIM; median: −.13, 99% CI: −.30 to −.03) loudness measure. We would like to stress that this result was expected as we by definition selected the most effective stimulation protocols. However, by including this analysis we could in a next step derive the signatures in oscillatory activity, which were related to tinnitus relief. Importantly, for every patient, we could identify a ‘real’ TMS protocol that was better at reducing tinnitus compared to the placebo sham stimulation (according to TIM scores). Loudness reductions and a distribution of the contributing stimulation protocols are shown in Figure 4.

**Figure 5:**
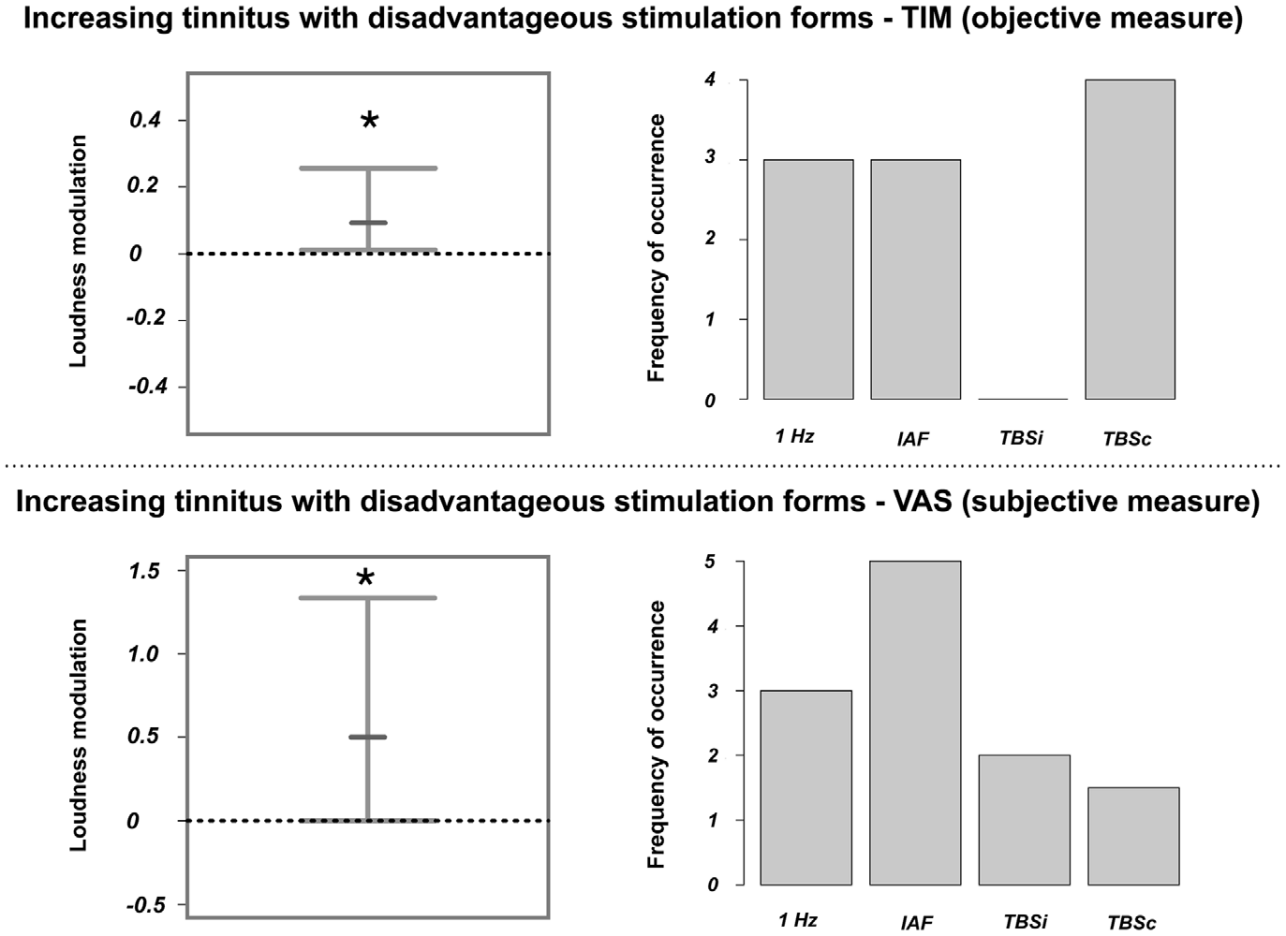
Changes in tinnitus loudness after application of the stimulation protocol that enhanced tinnitus loudness. The upper panel displays tinnitus loudness modulations that were measured with the tinnitus intensity matching procedure (TIM), while the lower panel illustrates tinnitus loudness modulations that were quantified using a visual analogue scale (VAS). The 95% confidence intervals are shown on the left side. The small bars display the median. The asterisks indicate significant modulations. As expected tinnitus loudness was significantly reduced after application of the individually selected protocol that worsened tinnitus. The distribution of these protocols is displayed on the right side. Note that, as the ambiguous cases (when selecting the most effective protocol with VAS) were included for this illustration, the summed frequency of occurrence can be higher than the total number of patients.

Analogously, we selected the stimulation protocols that consistently increased objective tinnitus loudness (TIM; median: .10, 99% CI: .01 to.24). It should be noted that as we could not select the stimulation protocols that enhanced tinnitus loudness unambiguously with the VAS scores for the majority of patients (only possible in 4 of 10 patients), we disregarded these values in further analyses. The loudness increase and a distribution of the contributing stimulation protocols are illustrated in Figure 5.

**Figure 6:**
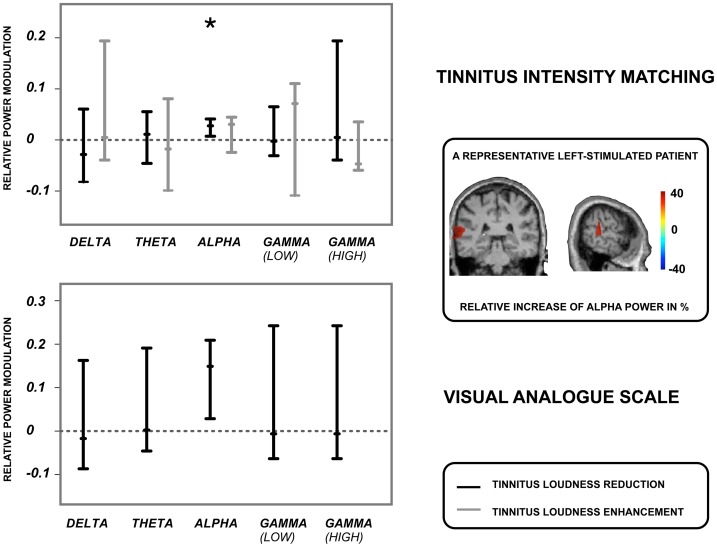
Modulation of oscillatory activity for the most effective TMS protocols. Shown are changes in oscillatory activity at the stimulated auditory cortex after application of the rTMS protocols that were best in reducing (black bars) and enhancing (grey bars) tinnitus loudness. The upper panel displays power modulations that were associated with the tinnitus intensity matching procedure (TIM), while the lower panel illustrates power modulations that were related to the visual analogue scale (VAS). Displayed are the 95% confidence intervals for power modulations in the stimulated auditory cortex and the different frequency bands (delta, theta, alpha, low gamma, high gamma). The small bars show the median, while the asterisk indicates the power modulations as significant. Note that we observed too many ambiguous cases for the VAS with respect to an increase in tinnitus loudness; we could thus not specify the according signature in oscillatory activity. Alpha power was significantly enhanced when tinnitus loudness was most effectively reduced by rTMS.

We could not identify any signatures of oscillatory activity in the stimulated auditory cortex related to a strong *increase* in tinnitus loudness (see Figure 6). However, when looking at the modulations of oscillatory power that were associated with a strong tinnitus loudness *reduction,* it turned out that a significant power enhancement in the *alpha* band was related to the tinnitus reduction (TIM alpha: median: .03, 95% CI: .01 to.04, VAS alpha: median:.15, 95% CI: .03 to.21). Delta, theta and gamma (low and high) power were not consistently modulated and varied strongly (confidence intervals included zero). This was true for both, subjective tinnitus loudness (VAS) in the six patients that could be unambiguously assigned to one stimulation protocol as well as objective tinnitus loudness (TIM) (see Figure 6 for comparison).

### 5. Auditory Alpha Power Modulation for the Individual Patients

**Figure 7:**
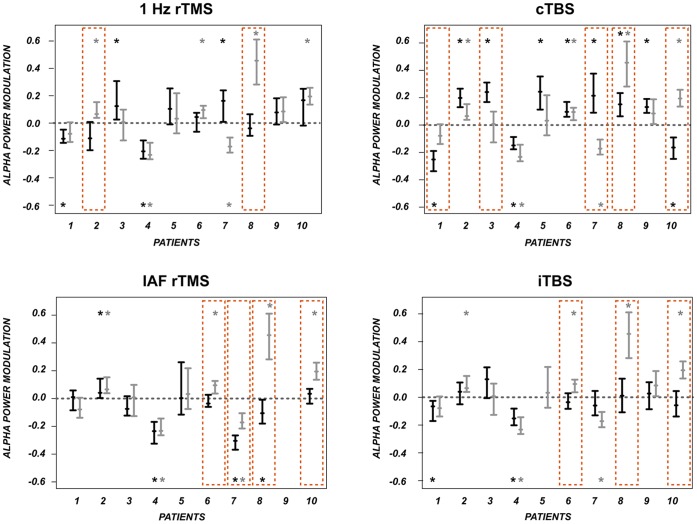
Modulation of auditory alpha power in the individual patients. Depicted are modulations of auditory alpha power in the individual patients and for the four active TMS protocols (black bars) and sham (grey bars). Shown are the 95% confidence intervals (uncorrected). The small bars display the median. The asterisks indicate significant modulations from pre to post TMS. The orange boxes point to significant modulations against sham stimulation. TMS modulates alpha activity significantly already in individual patients: 1 Hz rTMS (4 of 10, against Sham: 2 of 10), cTBS (10 of 10, against sham 5 of 10), IAF rTMS (4 of 10, against sham 4 of 10), iTBS (2 of 10, against Sham 3 of 10), however, not consistently into the same direction (increase vs decrease of alpha power).

Following the group results that showed a big variance within the stimulation protocols concerning tinnitus loudness modulations and also oscillatory activity modulations together with a potential key role of enhancing auditory alpha power in reducing tinnitus loudness (see section above) it is now depicted how the *individual* patients reacted to the different stimulation protocols. We thereby focussed on auditory alpha power. This statistic should be interpreted at an explorative level only as we did not control for multiple comparisons. The single subject statistics point to a high interindividual variability within the stimulation protocols. Overall, each stimulation protocol had a significant effect on auditory alpha power for a *subgroup* of the patients (95% confidence interval not including zero). In detail, alpha power was significantly modulated after application of 1 Hz rTMS in 4 of 10 patients (against sham 2 of 10), after IAF rTMS for 4 of 10 patients (against sham: 4 of 10), after cTBS in 10 of 10 patients (against sham: 5 of 10) and after iTBS in 3 of 10 patients (against sham: 3 of 10). However, the direction of the effects was not consistent resulting in the high variance and the lack of an effect on group level. This leads to the important conclusion that the variance is not due to individual participants with extreme values (this risk was already reduced by choosing the median), but that the effects of specific stimulation protocols on different patients are indeed highly variable. An overview of the auditory alpha power modulations for the single patients is given in Figure 7.

**Figure 8:**
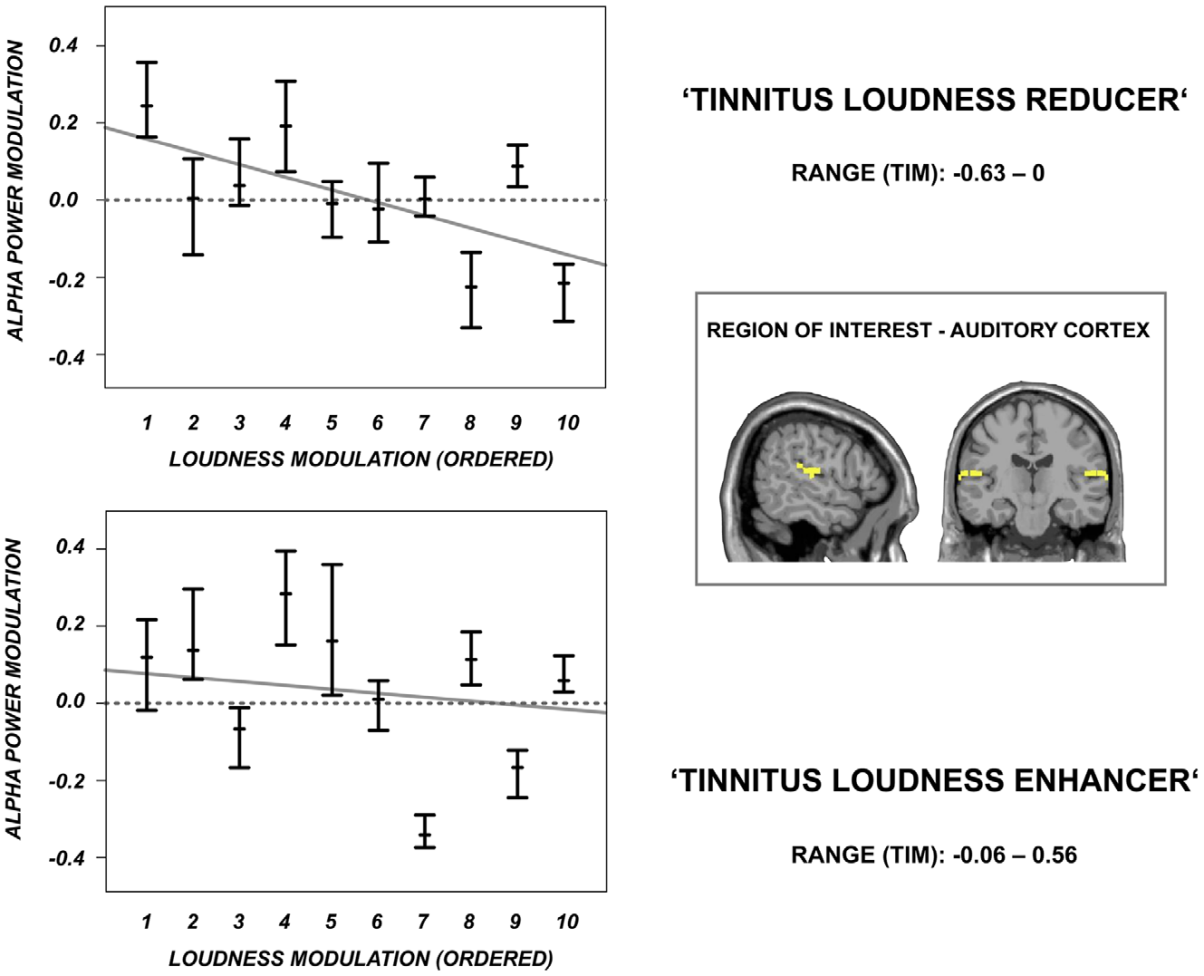
Modulation of oscillatory activity for the most effective TMS protocols and the individual patients. Shown are the changes in oscillatory activity at the stimulated auditory cortex for the individual patients after application of the rTMS protocols that were best in reducing (upper panel) and enhancing (lower panel) tinnitus loudness. Shown are the 95% confidence intervals for the individual patients. The small bars display the median. Patients are ordered according to the strength of the tinnitus loudness decrease (upper panel) or increase (lower panel). The grey line illustrates the correlation between the extent of the alpha power modulation and the extent of the tinnitus loudness modulation (ordered from 1 to 10). When tinnitus loudness is maximally reduced (range of TIM values: −0.63–0) the extent of the alpha power modulation is negatively correlated with the extent of the loudness reduction (RHO: –.61, p<. 05), thus the patients with the strongest alpha power increase were the patients with the strongest tinnitus loudness decrease. In contrast, When tinnitus loudness was maximally increased no such correlation was evident (RHO: –.37, p not significant).

To investigate if the association between auditory alpha power modulations and tinnitus loudness modulations is also visible on a single subject level we investigated the auditory alpha power modulations for the individually most effective stimulation protocol in reducing tinnitus loudness for the individual patients separately (as done before on a group level). We thereby disclosed an interesting pattern of auditory alpha power modulations for the ‘tinnitus reducers’: In particular the patients with a very strong tinnitus loudness reduction showed a strong auditory alpha power increase. The correlation between the individual auditory alpha power increase and the concomitant tinnitus loudness decrease was significant (Spearman’s rank correlation rho = –.61, p<.05). In contrast, the stimulation protocols that were most effective in enhancing tinnitus loudness revealed a rather inconsistent pattern (Spearman’s rank correlation rho = –.37, p not significant) what is in line with the inconsistent auditory cortex group results for the ‘tinnitus enhancers’. The individual alpha power modulations related to a strong reduction (and enhancement) of tinnitus loudness are displayed in Figure 8.

### 6. Signatures of Non-auditory Oscillatory Power that Result in a Strong Tinnitus Loudness Modulation

**Figure 9:**
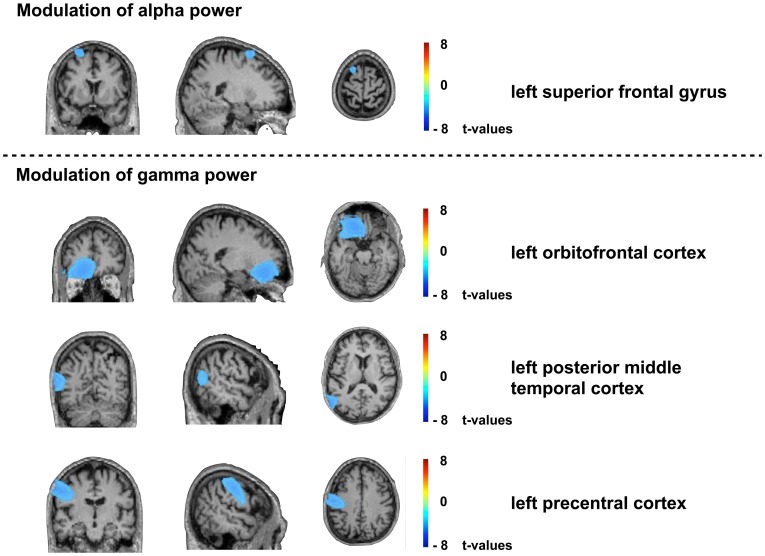
Changes in oscillatory activity in non-auditory brain areas for the most effective stimulation protocols. Shown are the changes in oscillatory activity in non-auditory brain areas after application of the rTMS protocols that were most effective in enhancing tinnitus loudness. The upper panel displays brain regions with power modulations in the alpha band, while the lower panel illustrates the areas exhibiting modulations in gamma power. Displayed are comparisons from pre to post rTMS and are quantified in t-values. Gamma power was significantly (p<.01; corrected) reduced in a left prefrontal, a left precentral and a left parieto-temporo-occipital region. Alpha power was reduced (p<.01; corrected) in a left superior frontal area.

We did not reveal any consistent non-auditory modulations of oscillatory activity associated with a strong tinnitus loudness decrease. However, we could identify left-hemispheric dominant reductions in oscillatory activity related to an increase in tinnitus loudness. Gamma power was significantly (p<.01, corrected) reduced in a left prefrontal (ventromedial frontal), a left precentral and a left parieto-temporo-occipital region. Furthermore, alpha power was reduced (p<.01, corrected) in a left superior frontal area. The power modulations in non-auditory regions related to a tinnitus worsening are displayed in Figure 9.

## Discussion

In the current study, we show how oscillatory brain activity is modulated in tinnitus patients by the application of four different TMS protocols (compared to sham) that are currently explored for treatment. We first focused on short-term modulations of oscillatory activity and tinnitus loudness that were consistent across patients for the specific TMS protocols, though overall effects were relatively small and varied strongly across patients. In a second step we identified the most effective stimulation protocols in decreasing (and increasing) tinnitus loudness and looked at associated modulations of oscillatory activity in auditory and non-auditory brain regions. As the increase of auditory alpha power turned out to be the main factor being related to a reduction of tinnitus loudness and as the most efficient stimulation protocol differed across patients we added a single-patient auditory alpha power analysis to further corroborate at an explorative level how the individual patients reacted to the different rTMS protocols. As expected from the group results, there was a high interindividual variability of the different rTMS protocols on alpha power and, importantly, the extent of the auditory alpha power increase was crucial for the extent of the tinnitus loudness reduction. This finding is of direct clinical relevance, since it suggests the potential of auditory cortex alpha power as a predictor for treatment outcome on an individual level.

### 1. Consistent Modulations of Tinnitus Loudness

We demonstrate that 1-Hz rTMS most consistently reduced tinnitus loudness (measured with a subjective and objective measure) compared to sham. However, the effects were relatively small and varied across subjects. This is in line with current literature on rTMS in tinnitus treatment that reports an impact of repeated 1-Hz rTMS sessions at the auditory cortex, albeit with moderate effect sizes and great interindividual variability [26], [30], [44]. Regarding the subjective estimates of tinnitus loudness, we additionally observed a trend for an overall reduction in tinnitus loudness after cTBS and, furthermore, an increase in tinnitus loudness after IAF rTMS. Again results were only moderate and varied strongly across patients (significant only on a trend level). The trend of a tinnitus relief after cTBS is consistent with the few reports on this relatively new stimulation paradigm tested for the treatment of tinnitus [45], [46]. At first glance, the trend pointing to an average increase in tinnitus loudness after IAF stimulation was rather unexpected, as most studies using high-frequency rTMS demonstrated a transient reduction of the tinnitus percept in the majority of the patients [47]–[50]. However, these discrepancies were likely due to differences in the experimental designs. In most studies, alterations in tinnitus loudness were assessed immediately after rTMS, whereas we assessed changes about 10–15 minutes after rTMS. Moreover, the duration of stimulation may play a role [51] as we measured tinnitus loudness after the application of 1000 pulses, in contrast to a maximum of 200 pulses in all previous studies on tinnitus. Consistent with the present data, that in average point to a disinhibition of the auditory cortex and increased tinnitus loudness after prolonged high-frequency rTMS, studies in the motor system show increased excitability and facilitated motor responses after such an extensive stimulation [52]–[54].

### 2. Consistent Modulations of Oscillatory Activity

We would like to emphasize that the group effects on auditory oscillatory activity were in general very small and should be rather interpreted at an explorative level. Alpha power (8–12 Hz) was significantly reduced (uncorrected) after treatment with IAF rTMS and iTBS in the auditory cortex ipsilateral to rTMS. It has been demonstrated that a decrease in alpha power is linked to disinhibition, whereas an increase in alpha power reflects active inhibition of the underlying neuronal tissue [19], [21], [55]–[60]. Regarding oscillatory activity in tinnitus patients, it has been shown that tinnitus patients exhibit a reduced alpha peak compared to normal controls, which is putatively linked to reduced inhibition in the auditory cortex [10], [19]. Thus, we suggest that the decrease in alpha power after rTMS is related to a disinhibition and hence an increase in excitability of the stimulated auditory cortex.

To our knowledge, the impact of high-frequency rTMS or iTBS on *auditory* cortex excitability has not yet been investigated, despite the presence of the above-mentioned studies on tinnitus. Research in the motor system usually reports an enhancement in excitability after high-frequency (in the alpha range) rTMS and iTBS [29], [54], [61]–[63]. Therefore, the decrease in alpha power after rapid-rate rTMS and iTBS fits well into the literature and furthermore extends the role of high-frequency rTMS and iTBS in increasing excitability from the motor to the auditory system.

It has to be mentioned here that the described effects of reduced auditory alpha power after high-frequency rTMS and iTBS were only moderate. However, this weak (IAF rTMS, iTBS) or also absent (1 Hz rTMS, cTBS) group effects were not due to absent effects in the individual patients but rather to a great interindividual variability as reflected by the single-patient analysis. The most powerful stimulation protocol, in the sense of affecting brain activity in most patients, for instance was cTBS: 10 of 10 patients (5 of 10 when testing against sham) showed a significant modulation (uncorrected) of auditory alpha power after the application of cTBS, 3 of them reducing and 7 of them enhancing significantly auditory alpha power. For every stimulation protocol we could find such a subgroup of patients that showed a strong modulation of auditory alpha power, albeit the direction of the effect (decrease or increase) was rather inconsistent. This finding can explain the high interindividual variability in treatment effects observed in many clinical trials [27], [30] on a neuronal basis and puts into question the possibility to develop a ‘standard therapy’ for the treatment of tinnitus. As the different stimulation protocols had specific effects on brain activity dependent on the individual patient, different patients may profit from different stimulation protocols. However, this assumption has still to be tested more conclusively by assessing the re-test reliability of the results.

High gamma power (70–90 Hz) was consistently modulated after two of the 4 active rTMS protocols: we detected a significant reduction (uncorrected) after 1-Hz rTMS as well as after iTBS in the stimulated auditory cortex. In contrast to the alpha rhythm, gamma oscillations are associated with higher-order functions and active sensory processing [64],[65]. It has been demonstrated that tinnitus patients exhibit enhanced auditory gamma power compared to controls [7], [20], [25] and that auditory gamma activity is also increased during transient tinnitus after noise trauma [17]. Furthermore, gamma power in the auditory cortex contralateral to the tinnitus percept has been suggested to reflect the loudness of the tinnitus percept [18].

Several studies have investigated the impact of low-frequency rTMS (≤1 Hz) on neuronal and behavioural outcomes. It has been demonstrated that low-frequency rTMS decreases cortical excitability [28], [61] (for an overview see [66]), reduces gamma activity in schizophrenic patients [67], improves inhibitory function in tinnitus patients [26], [27], reduces auditory metabolic activity in tinnitus patients [68] and reduces tinnitus loudness when applied during repeated sessions [26]. This is consistent with results from the present study that demonstrate a decrease in gamma power in the stimulated area and thus a reduction in auditory cortical activity after 1-Hz rTMS.

The effects of iTBS are rather variable and inconsistent regarding different stimulation areas: iTBS has been related to an enhanced motor cortex excitability [62] and an increased gamma power in the sensory-motor cortex of rats [69] which is in opposition to our finding of reduced gamma power after iTBS. However, studies also report an increased cortical inhibition [69], [70] as well as a reduction in the auditory steady-state response after iTBS [34]. In general, reports on the effects of iTBS on excitability of cortical regions apart from the human motor cortex are rare and suggest that the effects are not simply transferable to non-motor brain regions [71], [72]. The only study investigating the effect of iTBS on tinnitus loudness did not reveal any consistent effects [45]. Furthermore, a reduction in auditory gamma activity after iTBS does not necessarily point to a generally reduced excitability since we also detected a decrease in alpha power (rather pointing to increased excitability) following iTBS as described above.

All these inconsistencies in sum, we emphasize that the relationship of alpha power, gamma power and the perception of tinnitus loudness is not sufficiently understood to date and would require further research. In the following section we, however, attempt to further enlighten the role of auditory alpha power as a prerequisite for a change of tinnitus perception.

### 3. Signatures of Oscillatory Activity Associated with a Reduction in Tinnitus Loudness after rTMS in Auditory Brain Regions

As expected tinnitus loudness was most effectively reduced by individually selected stimulation protocols implying that different patients profited from different protocols. Importantly, for every patient, sham stimulation was worse at reducing tinnitus loudness than the best ‘real’ rTMS protocol. For about half of the patients 1 Hz rTMS was most successful while the other half profited from other protocols such as IAF rTMS and cTBS. However, we would like to stress here that the identification of individually most effective specific stimulation protocols might have been confounded by spontaneous fluctuations in tinnitus loudness. Despite being possible, this is unlikely as sham stimulation in no case was identified as the most successful protocol. For definitively ruling out this alternative explanation assessment of test re-test reliability by further studies will be needed.

Having selected the most effective stimulation protocol for each patient, we were then able to elucidate if the different rTMS protocols having in common to effectively reduce tinnitus loudness also affect ongoing auditory cortical activity in a specific way. Our findings demonstrate that a strong reduction in tinnitus loudness was associated with an enhancement of alpha power in the stimulated auditory cortex (ipsilateral to rTMS), while delta, theta and gamma power were not consistently modulated and varied strongly. Therefore, we suppose that an increase in alpha power in the auditory cortex is crucial for the reduction of tinnitus loudness, whereas auditory delta, theta and gamma power seem to be related to more unspecific effects. This is in line with studies that have demonstrated a normalisation in alpha power after successful tinnitus treatment using a neurofeedback approach [73], [74]. Importantly, the specific role of auditory *alpha* power does not contradict the results on the *average* modulations of tinnitus loudness and oscillatory activity described in the first part of the discussion where on average effective stimulation protocols (such as 1 Hz rTMS) were not associated with increases in auditory alpha power. Due to the fact that within categories patients that profit and do not profit from a specific protocol are automatically intermingled such an analysis is less sensitive in detecting tinnitus relief specific modulations than an individualised analysis. It rather focuses on modulations that are specific for the selected stimulation protocol regardless of its potential to strongly reduce tinnitus. We therefore think that the individualised analysis is more powerful to derive the features associated with tinnitus relief. It suggests the importance of alpha power increases in the auditory cortex as such a relevant feature to effectively reduce tinnitus.

Notably, due to the correlational approach of the current study we are not able to draw any conclusions about the directionality of the effects. Thus further studies are needed to clarify whether an increase in auditory alpha power indeed causes tinnitus relief, whether it rather reflects reduced auditory processing when tinnitus is reduced spontaneously or whether both effects are caused by a third mechanism.

We would like to point out that most studies on signatures in oscillatory activity related to tinnitus include comparisons between tinnitus patients and normal hearing controls [7], [10], [22], [23], [25]. The observed differences in neuronal activity are therefore not unequivocal and could be due to many unspecific mechanisms appearing in the tinnitus patients related to emotional or cognitive processes such as attention and evaluation. It is thus of great interest to identify the specific neuronal signatures in tinnitus patients (in this case oscillatory activity) that are related to a strong decrease (or increase) in tinnitus loudness compared to the ‘normal’ tinnitus perception and to find out which of these signatures must be modulated in order to successfully reduce tinnitus [88]. With respect to our data, we again emphasise the importance of an alpha power increase in the auditory cortex for an alleviation of the tinnitus percept. This is further substantiated by the single-patient auditory alpha power analysis showing that the patients with the strongest tinnitus loudness decrease were also the patients with the strongest alpha power increase. The association between high auditory alpha power and tinnitus relief further corroborates the role of alpha power in the active inhibition of cortical brain regions [19], [21], [56], [58], [60] and extends its role in the pathophysiology of brain diseases with an excitatory-inhibitory imbalance such as tinnitus [1], [10]. As tinnitus is predominantly characterised by a hyperexcitation in auditory brain regions [30], we suggest that enhancing auditory alpha activity is most relevant for the relief of tinnitus, putatively by increasing ongoing inhibitory mechanisms.

### 4. Signatures in Oscillatory Activity Associated with an Increase in Tinnitus Loudness after rTMS in Non-auditory Brain Regions

In this study we were not able to identify any extra-auditory signatures in oscillatory activity related to tinnitus loudness reduction. However, we found that an increase in tinnitus loudness was associated with a decrease in gamma and alpha power predominantly in left frontal regions. Specifically, tinnitus loudness increases were related to reduced alpha power in the left superior middle frontal region, which is in line with findings of increased activity in middle frontal and superior frontal regions in tinnitus patients [75]. The increase in tinnitus was further associated with gamma power reductions in the left prefrontal, left precentral and left posterior temporal cortex pointing to a deactivation of these regions when tinnitus loudness increased. Various studies underpin the relevance of a frontoparietal network in tinnitus perception [76]–[79]. It has been postulated that the prefrontal cortex integrates sensory and emotional aspects of tinnitus [8] and is part of a network associated with conscious tinnitus perception [10], [79]. Transcranial direct current stimulation [80] and transcranial magnetic stimulation [81] of the prefrontal cortex led to a decrease in tinnitus intensity and distress. A deactivation of the left prefrontal cortex associated with reduced positive affect [82], [83] and an increase in pain perception [84] is in line with the worsening of the tinnitus found in the present study. The precentral region has been related to the attentional control for the selection of auditory stimuli [85] and parieto-temporo-occipital regions were active during verbal auditory hallucinations [86].

However, the relevance of the observed top-down network including left frontal and centro-parietal regions for the generation of tinnitus and the significance of reduced alpha and gamma power in the sense of activation and deactivation must be investigated through further studies. It should also be noted that the lack of effects in non-auditory regions related to tinnitus reduction might be biased by the fact that the intervention was targeted at the auditory cortex. One may speculate that changes in tinnitus loudness through interventions focussing on other brain areas may reveal different patterns of network alterations.

### 5. Conclusion

The present results have shed further light on the pathophysiology of tinnitus and will hopefully stimulate the development of more effective therapy approaches. It appears essential to determine the signatures in auditory and non-auditory brain regions that are associated with the tinnitus percept in order to better understand this complex disease and to be able to develop more effective treatments. In this study we focused on the relationship of tinnitus loudness changes and changes in oscillatory activity in the stimulated auditory cortex and in other cortical regions. Our study confirmed that a reduction in tinnitus loudness is possible using conventional rTMS approaches; however, in line with most other studies, the general relief of the tinnitus percept was small and varied strongly across patients [27]. This pattern was also reflected in the neurophysiological data.

Beyond that we identified the signatures in oscillatory brain activity that relate to tinnitus relief. *Reductions* in the tinnitus sensation were associated with increases in alpha power in the stimulated auditory cortex, meaning that the intervention had specific effects. The identification of alpha power increases in the stimulated area as the relevant mechanisms of action for tinnitus relief is of high relevance as it provides an initial orientation for an individualized treatment approach. Future clinical studies may aim at identifying the optimal rTMS protocol having the potential to reliably increase alpha activity in the temporal cortex in the individual patient in order to enhance clinical efficacy. On the other hand, *increases* in the tinnitus sensation were related to alterations in a left-lateralised fronto-centro-parietal network, confirming the relevance of this network for tinnitus perception. Increase in tinnitus loudness may thus either result from propagated rTMS effects on non-auditory (mostly left frontal) brain regions or may be unspecific. More comprehensive clinical trials are needed in order to further explore the observed effects of temporal rTMS on cortical oscillations in tinnitus patients, regarding in addition to this clinical relevance and the persistence of these effects.

## Supporting Information

Figure S1 **Distribution of extreme values, exemplary for alpha power modulations.** The upper panel illustrates a boxplot distribution of the data for the different rTMS protocols. Extreme values were detected after cTBS, IAF rTMS and iTBS, and not after 1-Hz rTMS and sham. The lower panel illustrates the relation between tinnitus duration and auditory alpha power modulation. Extreme values are exclusively associated with very short tinnitus duration.
(PDF)

Figure S2 **Consistent changes in oscillatory activity after the four active TMS protocols (1 Hz rTMS, cTBS, IAF rTMS, iTBS) compared to sham.** The upper left panel displays delta power modulations, the upper right panel illustrates theta power modulations and the lower left panel shows modulations of low gamma power at the stimulated auditory cortex. The stimulated region and region of interest is displayed on the lower right side. Shown are the 95% confidence intervals. The small bars display the median. No significant power modulations were found either in the delta, theta or in the low gamma band.
(PDF)
