# Supplementary material for: rTMS Induced Tinnitus Relief Is Related to an Increase in Auditory Cortical Alpha Activity
Source: PLoS One. 2013 Feb 4;8(2):e55557. doi: 10.1371/journal.pone.0055557 (PMC3563643; doi:10.1371/journal.pone.0055557)
Supplement: Figure S1 — Distribution of extreme values, exemplary for alpha power modulations. The upper panel illustrates a boxplot distribution of the data for the different rTMS protocols. Extreme values were detected after cTBS, IAF rTMS and iTBS, and not after 1-Hz rTMS and sham. The lower panel illustrates the relation between tinnitus duration and auditory alpha power modulation. Extreme values are exclusively associated with very short tinnitus duration. (PDF) [file pone.0055557.s001.pdf]

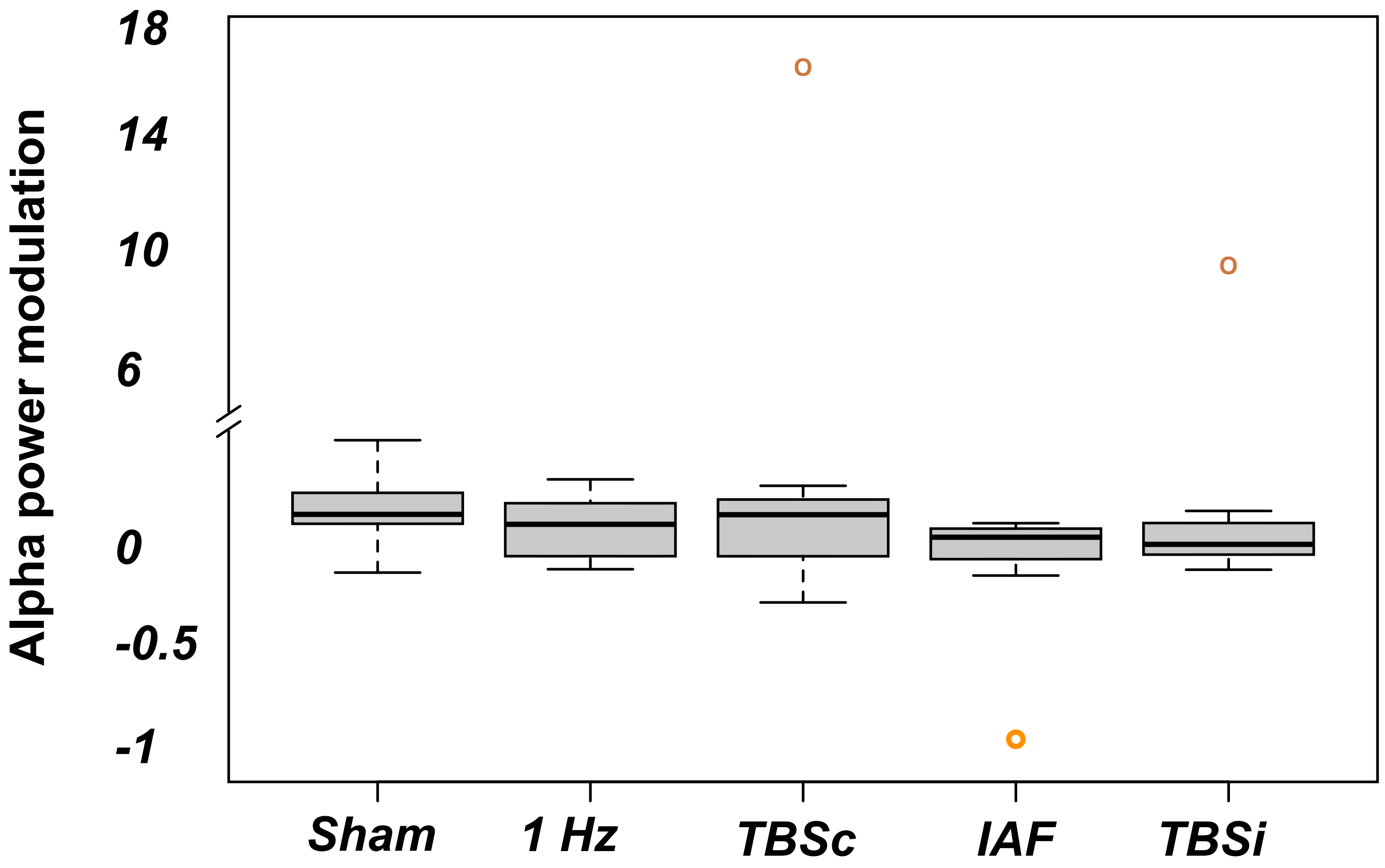

Boxplot distribution for the different TMS protocols

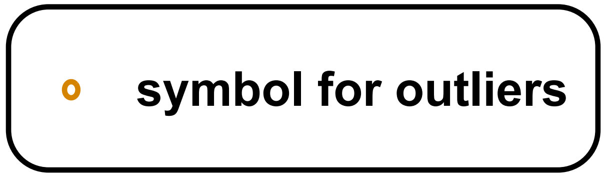

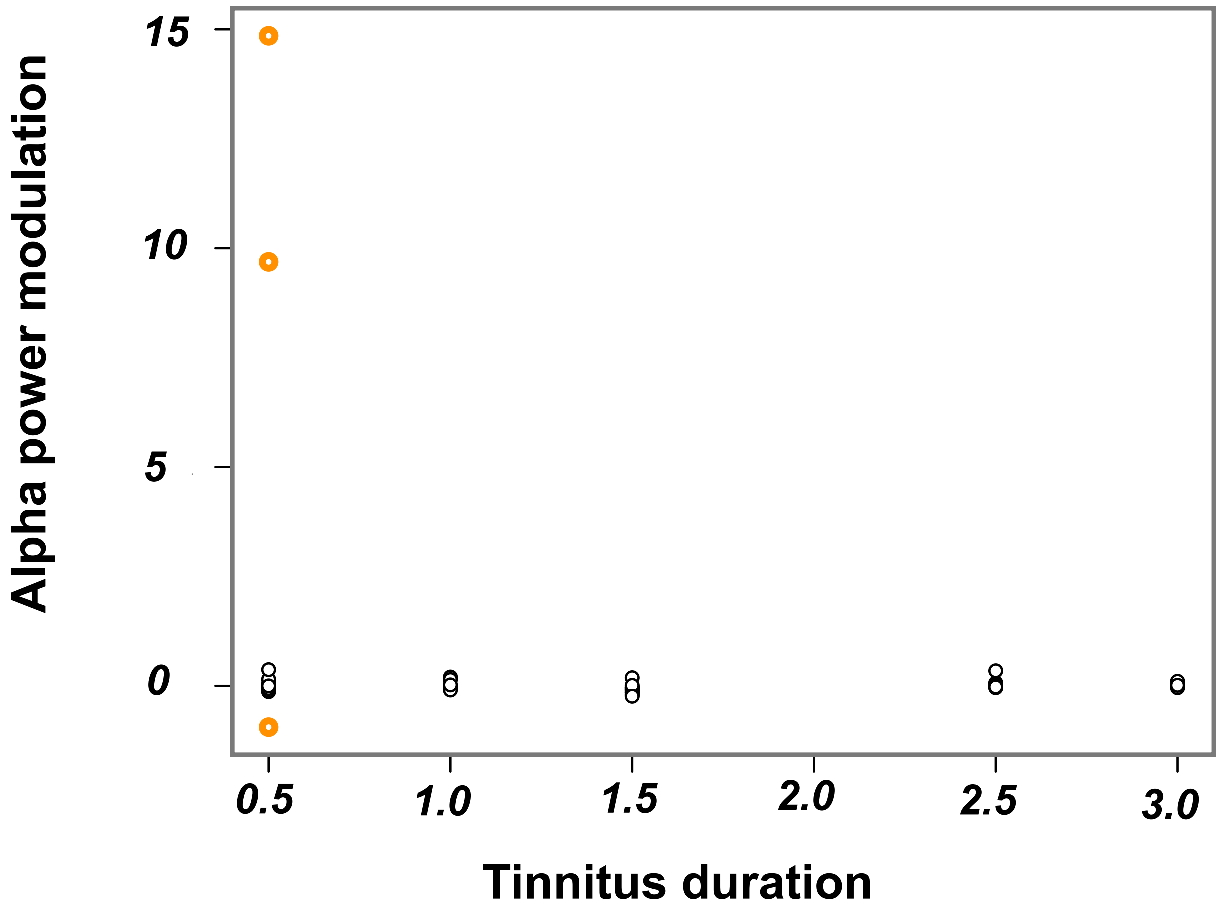

Relation between tinnitus duration and alpha power modulation
