# Supplementary material for: rTMS Induced Tinnitus Relief Is Related to an Increase in Auditory Cortical Alpha Activity
Source: PLoS One. 2013 Feb 4;8(2):e55557. doi: 10.1371/journal.pone.0055557 (PMC3563643; doi:10.1371/journal.pone.0055557)
Supplement: Figure S2 — Consistent changes in oscillatory activity after the four active TMS protocols (1 Hz rTMS, cTBS, IAF rTMS, iTBS) compared to sham. The upper left panel displays delta power modulations, the upper right panel illustrates theta power modulations and the lower left panel shows modulations of low gamma power at the stimulated auditory cortex. The stimulated region and region of interest is displayed on the lower right side. Shown are the 95% confidence intervals. The small bars display the median. No significant power modulations were found either in the delta, theta or in the low gamma band. (PDF) [file pone.0055557.s002.pdf]

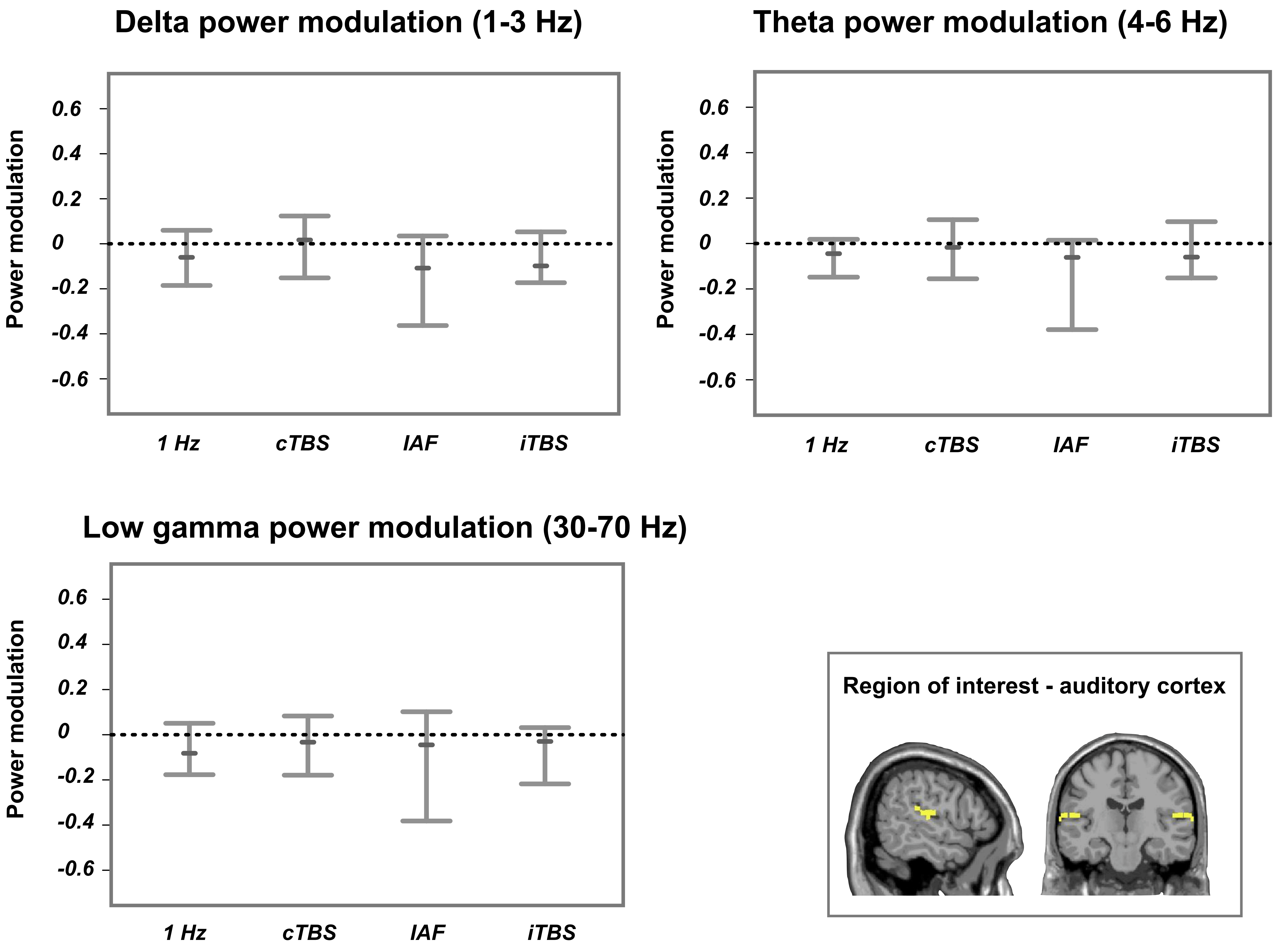

Delta power modulation (1-3 Hz)
Theta power modulation (4-6 Hz)
Low gamma power modulation (30-70 Hz)
Power modulation
0.6
0.4
0.2
0
-0.2
-0.4
-0.6
1 Hz
cTBS
IAF
iTBS
Region of interest - auditory cortex
